# New York State and Tuberculosis in Cattle of the United States

**Published:** 1892-11

**Authors:** 


### NEW YORK STATE AND TUBERCULOSIS IN CATTLE OF THE UNITED STATES.

New York is the first, we believe, to undertake the systematic eradication of tuberculosis among its herds.

At the last session of the legislative assemblies of this State there was passed and approved by the Governor, May 5, 1892, "An Act in relation to tuberculosis in milch cows and other cattle and infectious and contagious diseases of cattle." This Act forms Chapter 487 of the Laws of 1892.

The Act places the work under the supervision of the State Board of Health and directs it to use all reasonable means for ascertaining the existence of diseases and for preventing all injury from tuberculosis in milch cows; and to take measures to suppress this disease promptly.

The Act contains clauses enabling inspectors appointed under its provisions to call upon the sheriff in the case of interference with duties, and providing for the indemnity to the owner for slaughtered animals of the actual value at the time they are killed.

For carrying out its provisions, five thousand dollars are appropriated. The payment for slaughtered cattle is to be settled by the court of claims and apparently to be paid out of the general fund.

This is in brief the tenor of the Act before us. During last September, Doctor Austin Peters was appointed Superintendent of Investigations in Tuberculosis, and Dr. Cooper Curtice a Cattle Inspector, by the Board of Health. With this small force they have begun and are carrying out field work in Westchester County.

With the amount of money at their disposal, much positive good in the way of ridding New York from tuberculosis by the Board of Health can not be expected. But the sum should be amply sufficient for prosecuting the work on the small scale outlined and the extermination of all determinable cases of the disease within Westchester county or such part of it as can be inspected. With positive results obtained, both from inspection and from extermination, the Board of Health should be able to go before the committees at the next assembly at Albany and obtain from them an appropriation more nearly commensurate with the magnitude of the work for its continuation.

To veterinarians, the cause in which the Board of Health is engaged needs no advertising. The president, Mr. T. H. Newbold, and the secretary and executive officer, Dr. Lewis Balch, of the Board, deserves much praise from the profession for the active interest they take in this matter and for securing the passage of the present bill, which, though inadequate, is a long step ahead.

The readers of this Journal can assist the enterprise by sending to the Board of Health at Albany reports of herds infected with tuberculosis in any county of New York State. Such reports, even if not investigated at once, could be compiled to show the positive occurrence of the disease as scattered throughout the State.

We shall look forward with interest to the final report of the Board of Health and to the outcome of its action in Westchester County.

